# Synthesis and performance of ZnO quantum dots water-based fluorescent ink for anti-counterfeiting applications

**DOI:** 10.1038/s41598-021-85468-z

**Published:** 2021-03-12

**Authors:** Xi Chen, Qi Wang, Xiao-Ju Wang, Jie Li, Guo-Bin Xu

**Affiliations:** College of Light Industry and Food Engineering, Nanjing Forestry University, Nanjing, 210037 China

**Keywords:** Chemical engineering, Applied optics, Materials for optics, Nanoscale materials, Other nanotechnology, Biosynthesis, Materials chemistry, Photochemistry

## Abstract

In this study, the ZnO quantum dots (QDs) water-based fluorescent anti-counterfeiting ink was prepared with the polyvinylpyrrolidone (PVP) content of 0.15–0.17 g/mL, the ZnO QDs concentration of 4% and water as the solvent, which has good fluorescence, printability and resistance. According to the halftone technology, fluorescence quenching of the ZnO QDs by acid, and acid resistance of the organic fluorescent ink, a high-quality anti-counterfeiting method of fluorescent discoloration was proposed. The QDs ink has broad application prospects in the field of anti-counterfeiting green packaging.

## Introduction

Fluorescent anti-counterfeiting ink is one of the widely used printing anti-counterfeiting technologies. It has the characteristics of good concealment, low cost, and convenient identification. The traditional fluorescent inks are typically represented by three-primary-color organic fluorescent inks, which are widely used in banknotes, securities, certificates, bills and high-grade packaging^1–4^.With the in-depth development and popularization of nanotechnology, nanomaterials such as perovskite QDs^5,6^, II-VI semiconductor QDs^7–11^ and carbon-based dots^12–14^ can be used as new fluorescent pigments to prepare nano fluorescent ink for their excellent optical properties. Among them, ZnO QDs have large exciton binding energy. They are easy to achieve high-efficiency stimulated emission fluorescence at room temperature^15^, and different particle sizes can achieve different fluorescent colors^16–20^. ZnO QDs have high abundance in nature^21^, low cost of preparation, diverse and mature methods^22,23^, and there are many researches about improving the water solubility of ZnO QDs^18,20,24^.Moreover, ZnO QDs are mostly used in bioimaging^25,26^ and drug delivery^27,28^ with superior biocompatibility. Therefore, the application of ZnO QDs to environmentally friendly water-based fluorescent anti-counterfeiting ink has a solid research foundation. The anti-counterfeiting applications of fluorescent ink are mainly reflected in two ways. One is the display of colored fluorescence under ultraviolet light excitation^7,29^. The other is the fluorescence quenching and fluorescence recovery under external conditions^8,11^. These two methods focus on the properties of the fluorescent dyes themselves and fail to reflect the anti-copy function of the anti-counterfeiting method itself.

Herein, we used water-soluble ZnO QDs prepared by the sol–gel method as the fluorescent pigment, water as the solvent, and PVP as the viscosity modifier. The fluorescence and printability were used as evaluation indicators to explore the pH value, the concentration of ZnO QDs and the addition amount of PVP to prepare water-based fluorescent anti-counterfeiting ink. According to the principles of pre-press image processing and the fluorescence quenching of ZnO QDs by acid^21^, the anti-counterfeiting method of fluorescent ink was designed, and the effect was verified by screen printing. The multiple anti-counterfeiting properties of the method made it more security.

## Materials and methods

### Materials

Zinc acetate dihydrate (Zn(Ac)_2_·2H_2_O, AR), potassium hydroxide (KOH, AR), absolute ethanol (C_2_H_5_OH, AR), hydrochloric acid (HCL, AR) and hexane (CH_3_(CH_2_)_4_CH_3_, AR) were purchased from Sinopharm Chemical Reagent Co., Ltd. 3-aminopropyltriethoxysilane (APTES, purity 98%) and ammonia water (NH_3_·H_2_O, ACS) were purchased from Aladdin. PVP (MW ≈ 1,300,000) was purchased from Macklin. Vinegar (edible grade, Jiangsu Hengshun vinegar Co., Ltd) was purchased from a supermarket. Organic colorless-red fluorescent anti-counterfeiting ink (CR ink) that is colorless under visible light and red fluorescence under ultraviolet light was purchased from Aobo Security Technology Co., Ltd. Deionized water was used in all experiments.

### Synthesis and modification of ZnO QDs

The first step is to synthesis ZnO QDs. at 60 °C, 140 mmol KOH was dissolved in 80 mL ethanol, and then reduced temperature to 4 °C. 100 mmol Zn(Ac)_2_·2H_2_O was put into a flask with a 600 mL ethanol solution and kept stirring at 78 °C until dissolved. The KOH solution was added to the Zn(Ac)_2_·2H_2_O solution slowly, and reacted for 30 min to result in a ZnO QDs solution. Then the unmodified ZnO QDs (U-ZnO QDs) appeared by mixing the ZnO QDs solution with CH_3_(CH_2_)_4_CH_3_ at 1:2 volume ratio. The second step of modifying ZnO QDs was achieved by adding a mixed solution of 1.6 mL APTES and 6 mL deionized water dropwise to the ZnO QDs solution. After this, silane surface modified ZnO QDs (M-ZnO QDs) gradually all precipitated out.

Finally, the precipitate was centrifuged by a centrifuge (6000 rpm, 2 min) and the precipitate was washed fifth with ethanol to remove the unreacted precursors. The powders of the U-ZnO QDs and M-ZnO QDs were achieved by drying (60 °C, 6 h) the washed sediment and dissolved into water for further applications.

### Formulation of ZnO QDs water-based fluorescent ink

M-ZnO QDs were used as the fluorescent dye, water and PVP as the solvent and viscosity regulator. According to the specific proportion, keeping stirring and ultrasonic dispersion until they were uniformly mixed to make the ZnO QDs water-based fluorescent ink. Subsequently, the ink was printed onto the surface of the glazed printing paper that has a basis weight of 100 g using a 200-mesh screen printing plate and allowed to dry naturally.

### Characterizations

The structural properties of the ZnO QDs were characterized using a JEM-1400 transmission electron microscope (TEM) and a JEM-2100 UHR transmission electron microscope (HRTEM). The crystallinity of the ZnO QDs were studied using X-ray diffraction (XRD). The radicals in the ZnO QDs were characterized using a Bruker VERTEX-80 Fourier transform infrared (FTIR) spectrometer. The photoluminescence (PL) properties of the QDs were evaluated in a PerkinElmer LS55 spectrophotometer employing the 365 nm of a xenon lamp as the excitation source. The absorption spectra of the QDs were acquired on a TU-1900 double beam UV–vis spectrometer. The viscosity properties of the ink were measured by Brookfield DV-I+ Viscometer. The rheological properties of the ink were measured by a MARS60 rheometer. The colors were observed using Artificial Daylight 6500 K (D65) light and ultraviolet (UV) light in the CPC-7 standard light box.

## Results/discussion

### Structural characterization and fluorescence performance of the ZnO QDs

**Figure 1:**
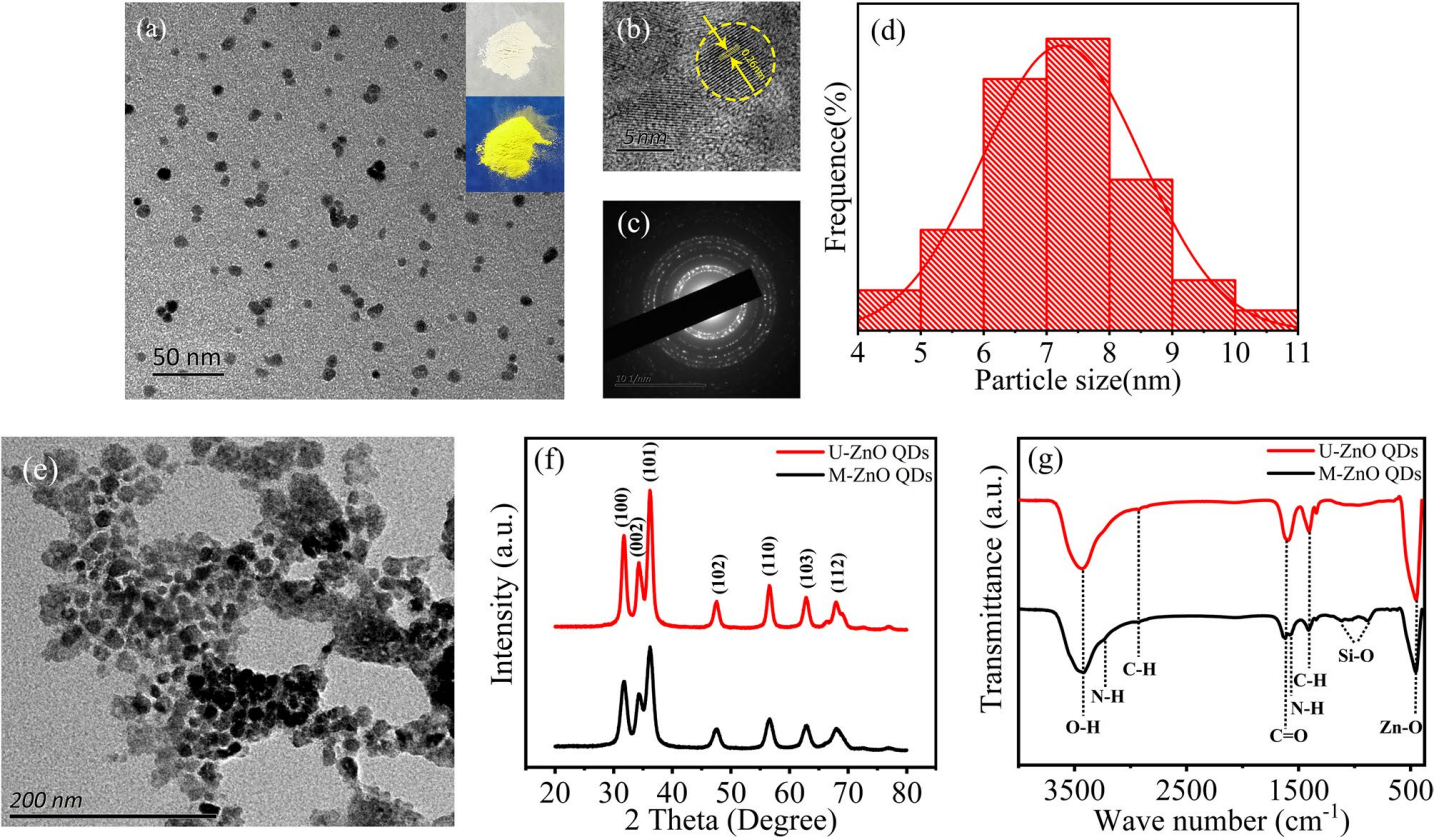
(**a**) A TEM image of M-ZnO QDs (The inset shows the effect of M-ZnO QDs powder under D65 light and UV light). (**b**) A HRTEM image of M-ZnO QDs. (**c**) A SAED pattern of M-ZnO QDs. (**d**) A histogram of the particle size distribution of M-ZnO QDs. (**e**) A TEM image of U-ZnO QDs (**f**) The XRD patterns of U-ZnO QDs and M-ZnO QDs. (**g**) The FTIR spectra of U-ZnO QDs and M-ZnO QDs.

The dispersion effects in water of M-ZnO QDs and U-ZnO QDs are shown in Fig. 1a and e. It is obvious that M-ZnO QDs have better dispersibility in water, have regular spherical shape and good dispersion, so they can be used as fluorescent pigment in water-based ink^30^. However, U-ZnO QDs have agglomeration and stacking phenomenon. The inset indicates that M-ZnO QDs powder shows bright yellow fluorescence under UV illumination. In Fig. 1b, clear lattice fringes with a spacing of 0.26 nm are matching the inter-planar distances of the (002) plane of wurtzite ZnO. It is confirmed that the crystal is ZnO crystal^20^. In Fig. 1c, the selected area electron diffraction (SAED) pattern shows seven clear rings corresponding to different crystal planes of ZnO, indicating that M-ZnO QDs have high crystallinity and physical stability. As shown in Fig. 1d, the diameter of M-ZnO QDs ranges from 4 to 11 nm and the average particle size is 7.4 nm, which is one of the factors that could ensure the great fluorescence properties of ZnO QDs^17,21,31–35^.

The purity of ZnO QDs will affect the ink performance. The higher the purity is, the better the quality of the ink is. As shown in Fig. 1f, there are 7 characteristic peaks corresponding to (100), (002), (101), (102), (110), (103) and (112) crystal planes respectively. It is proved that the samples are pure ZnO QDs. From the Scherrer equation^36^ that correlates the peak broadening with the quantum dot size, we have calculated M-ZnO QDs diameters of 7.4 nm that is the same with TEM results. The characteristic peak intensity of M-ZnO QDs is lower than that of U-ZnO QDs. Due to the modification of APTES, a coating has been formed on the surface of the ZnO QDs to weaken the diffraction peak intensity^37^.

The surface functional groups of the ZnO QDs have been characterized by FTIR. In Fig. 1g, for U-ZnO QDs, the Zn–O bond stretching vibration appears at about 454 cm^−1^. The absorption bands at about 1410 cm^−1^ and 2923 cm^−1^ could be attributed to the bending vibration and stretching vibration of C–H. The band at about 1619 cm^−1^ belongs to the C=O bond stretching vibration, and the peak at about 3420 cm^−1^ is due to the stretching vibration of O–H. In comparison, for M-ZnO QDs, the transmittance of Zn–O bond is weakened. The absorption bands at about 1113 cm^−1^ and 884 cm^−1^ could be attributed to the stretching vibration of Si–O. The peaks at about 1573 cm^−1^ and 3235 cm^−1^ are attributed to the bending vibration and stretching vibration of N–H. Thus, due to the hydrolysis of APTES, the ZnO QDs have been modified by silane, which can prevent the fluorescence quenching caused by agglomeration^37^. The solubility is attributed to the exposed hydrophilic groups (–NH_2_, –OH).

**Figure 2:**
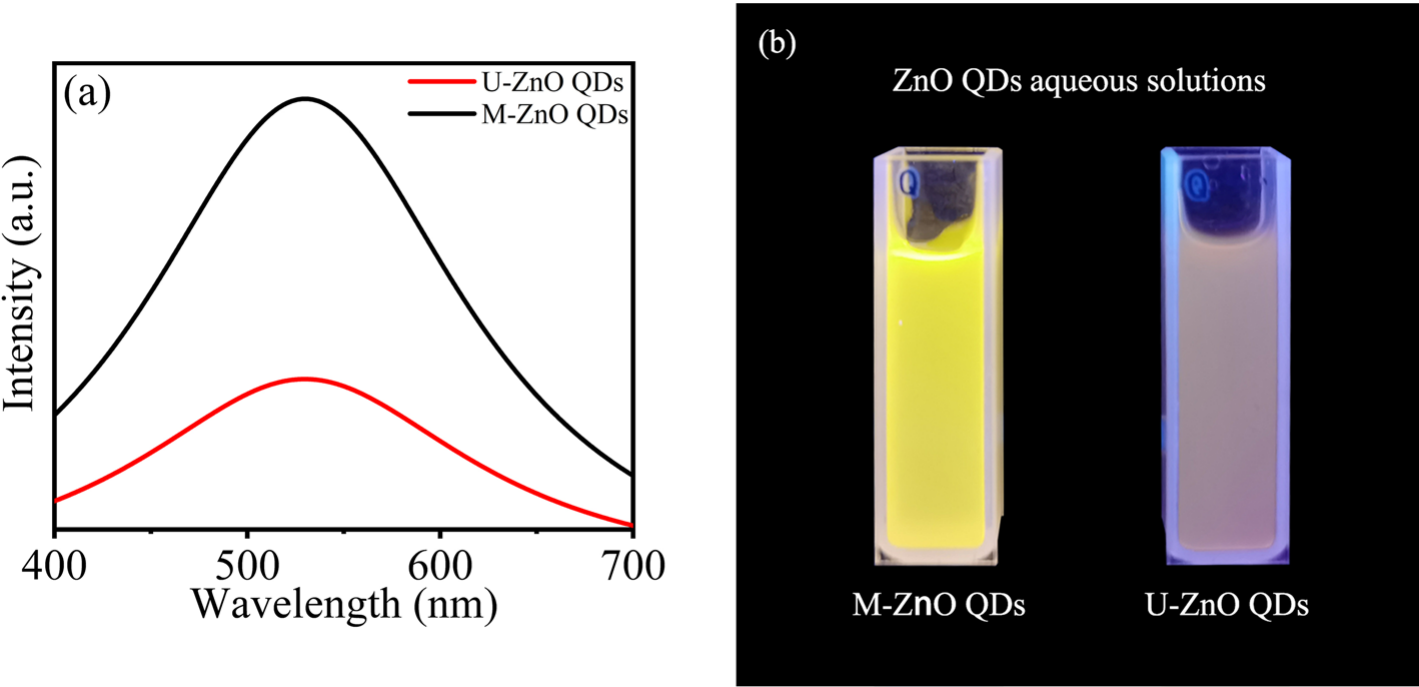
(**a**) The fluorescence spectra of U-ZnO QDs and M-ZnO QDs. (**b**) The fluorescence images of U-ZnO QDs and M-ZnO QDs.

Under the excitation of 365 nm UV light, as shown in Fig. 2a, the emission peaks of M-ZnO QDs and U-ZnO QDs aqueous solutions are both around 530 nm, but the intensity of M-ZnO QDs is almost three times that of U-ZnO QDs. Figure 2b can be intuitively observed that the fluorescence performance of M-ZnO QDs aqueous solution is better.

### Influencing factors on fluorescence performance of the aqueous ZnO QDs

**Figure 3:**
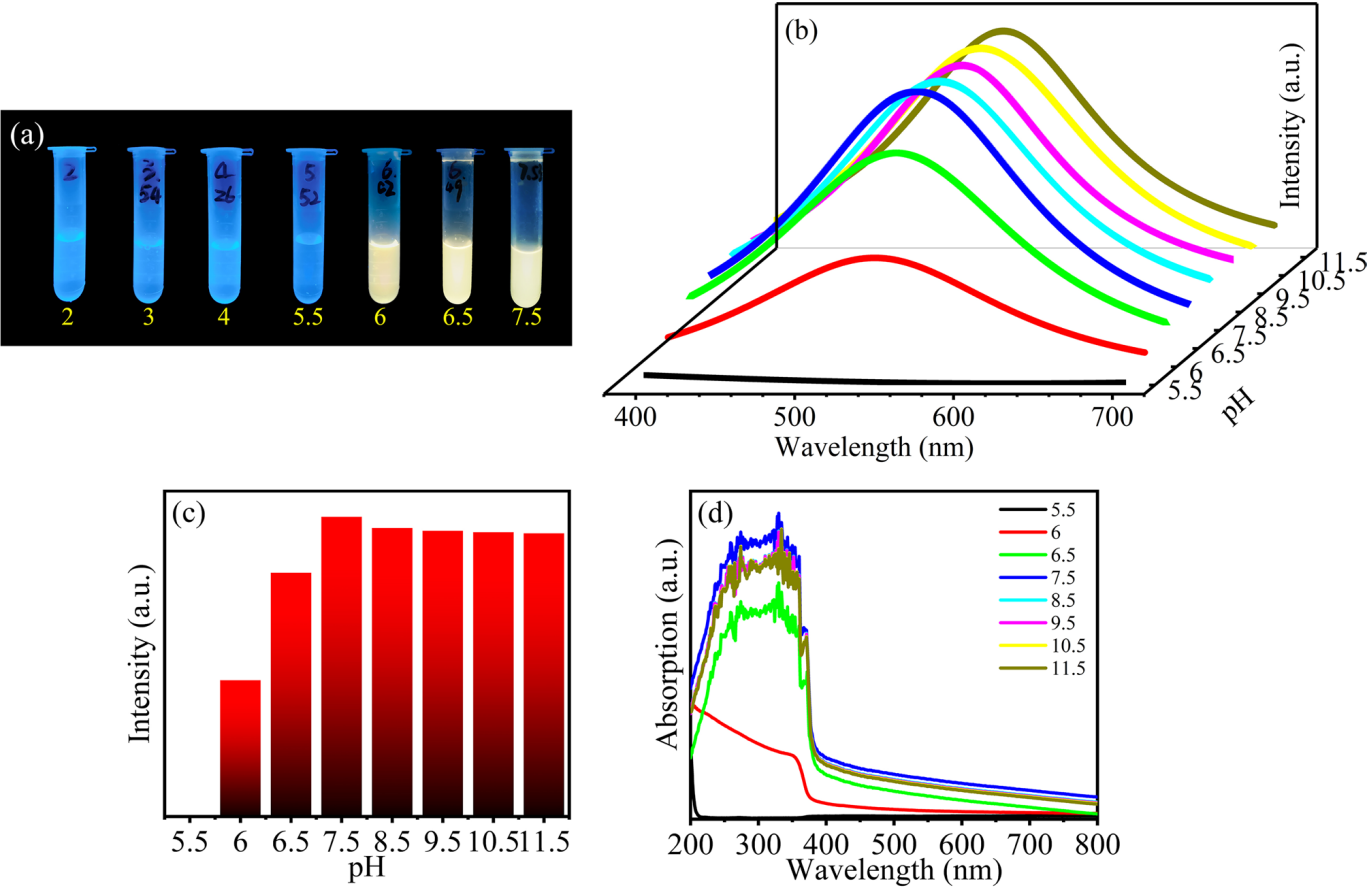
(**a**) The fluorescence images of M-ZnO QDs at different pH values. (**b**) The fluorescence spectra of M-ZnO QDs at different pH values. (**c**) The fluorescence peak histogram of M-ZnO QDs at different pH values. (**d**) The UV–vis spectra of M-ZnO QDs at different pH values.

The pH value affects the fluorescence performance of the ZnO QDs^11,38^. M-ZnO QDs solutions with pH 2–11.5 were prepared to explore the relationship between the two and the pH value was adjusted by HCL or NH_3_·H_2_O. The fluorescence images show in Fig. 3a. When the pH values are between 2 and 5.5, the solutions have no visible fluorescence. PH 6 is the critical point of fluorescence. After that the fluorescence enhances with the increase of the pH value. When the pH value is greater than 7.5, the fluorescence intensity decreases slightly. Figure 3b and c also show that the fluorescence intensity is the strongest at pH 7.5. When the pH value increases again, the fluorescence intensity decreases slightly, which is due to the agglomeration caused by increased attractive force between the ZnO QDs under alkaline conditions^39^. However, the fluorescence quenching occurs when the pH value is less than 6, which is caused by the decomposition of the QDs in an acidic environment^40^.The UV–vis spectra of M-ZnO QDs solutions are indicated in Fig. 3d. When the pH value is greater than or equal to 6, the solutions have certain absorbability in the ultraviolet region. When the pH value is 5.5, the QDs solution almost does not have absorption. Therefore, the pH 7.5 can be used as a reference for preparing fluorescent ink. Nevertheless, the acidic conditions with pH less than or equal to 5.5 can lead to the fluorescence quenching.

**Figure 4:**
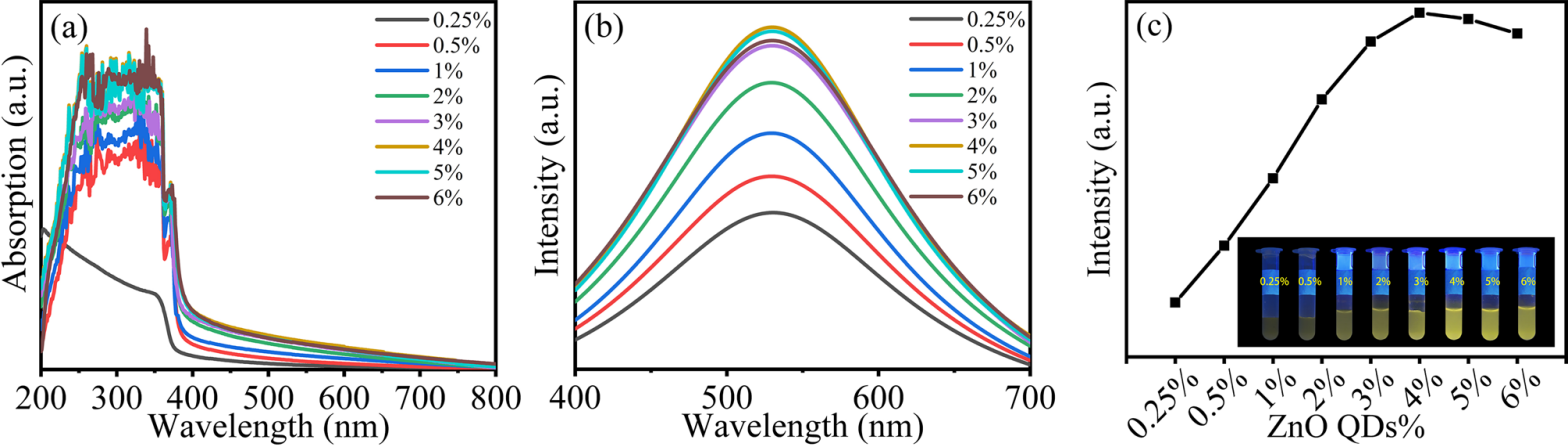
(**a**) The UV–vis spectra of M-ZnO QDs solutions with different concentrations. (**b**) The fluorescence spectra of M-ZnO QDs solutions with different concentrations. (**c**) The trend of fluorescence intensity of M-ZnO QDs solutions with different concentrations (the inset is the fluorescence effect of M-ZnO QDs solutions with different concentrations).

The concentration of the ZnO QDs in the aqueous solution also affects the fluorescence performance. As shown in Fig. 4a, the 0.25–6% M-ZnO QDs solutions have obvious absorption in the ultraviolet region of 200–400 nm. It gradually enhances with the increase of the concentration of M-ZnO QDs. When the concentration is greater than or equal to 4%, the UV absorption intensity reaches a high level and tends to stabilization. As indicated in Fig. 4b, the emission peaks are all broad peaks around 530 nm, which is due to the surface defects of the ZnO QDs^35^. The position of the fluorescence peak is not affected by the concentration of the ZnO QDs. When the concentration of the QDs is 0.25–4%, the fluorescence intensity increases obviously with the increase of the QDs concentration, reaching the strongest at 4%. When the concentration increases to 5–6%, the fluorescence intensity no longer increases and slightly decreases at 6%. Figure 4c illustrates the influence of the concentration of M-ZnO QDs on the fluorescence performance of the solutions more intuitively. When the concentration of M-ZnO QDs is greater than 5%, the fluorescence effect will be reduced due to the agglomeration of the QDs. To sum up, the concentration of M-ZnO QDs is 4%, which can be used as a reference for preparing water-based fluorescent ink.

### Formulation and performance of the ZnO QDs water-based fluorescent ink

Viscosity is an important indicator of ink stickability. PVP is a commonly used viscosity modifier in nano ink preparation^41^. This research is based on the viscosity range from 2000 to 6000 mPa s that is the standard of screen plate of ultraviolet fluorescent anti-counterfeiting printing ink in GB/T 17001.1-2011. On the premise of determining the pH value and concentration of the ZnO QDs, the synthesis of the screen-printing ink is completed by adding PVP.

**Figure 5:**
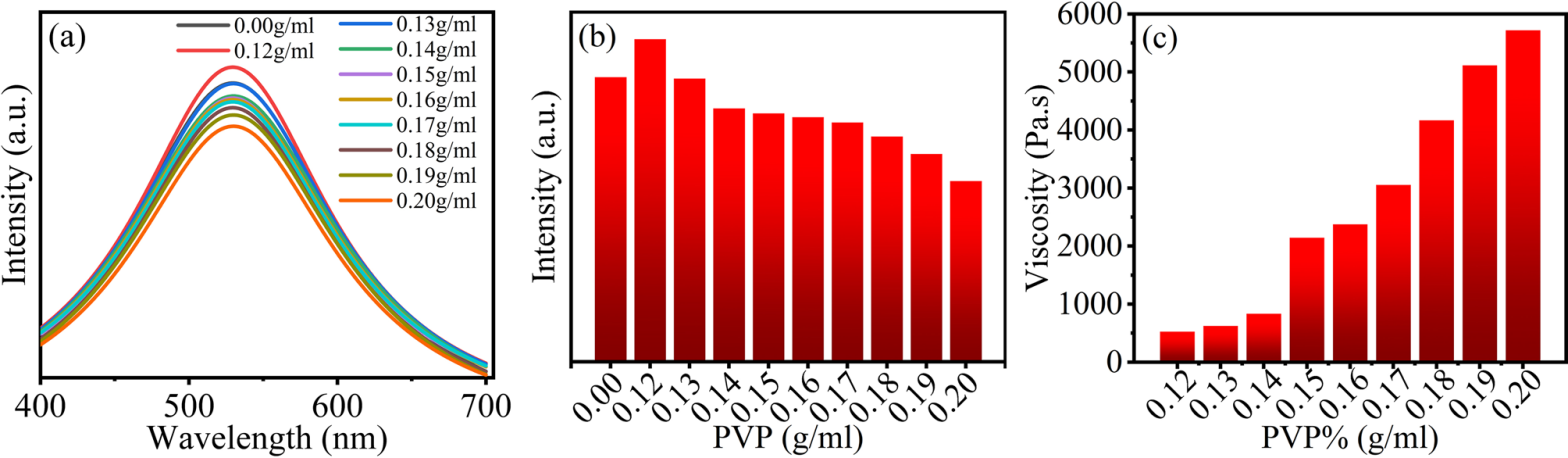
(**a**) The fluorescence spectra of the ZnO QDs inks with different PVP contents. (**b**) The fluorescence peak histogram of the ZnO QDs inks with different PVP contents. (**c**) The viscosity histogram of the ZnO QDs inks with different PVP contents.

Using the 4% M-ZnO QDs aqueous solution with the pH value of 7.5 as the basic reagent and reference sample, 9 groups of samples with the PVP content of 0.12–0.20 g/mL were prepared and their fluorescence performance and viscosity were tested. Figure 5a shows that the PVP content does not affect the position of fluorescence emission peak. The fluorescence intensity decreases with the increase of the PVP content. The fluorescence intensity of the PVP with 0.12 g/mL is better than the reference, because the addition of the PVP helps to improve the dispersion of the ZnO QDs. When the content of the PVP is 0.13–0.17 g/mL, the fluorescence intensity decreases slightly compared with the reference. When the amount of the PVP added is greater than or equal to 0.18 g/mL, the fluorescence intensity decreases significantly, which is due to the partial agglomeration of the QDs caused by the crosslinking action when the PVP is too much. Figure 5b can be observed the above conclusion directly. As indicated in Fig. 5c, when the addition of the PVP is greater than or equal to 0.15 g/mL, the viscosity of the ink can meet the requirements of GB/T 17001.1-2011. Combined with the influence of the PVP addition on the fluorescence effect of the samples, 0.15–0.17 g/mL is the optimal addition amount of PVP in the ZnO QDs ink.

**Figure 6:**
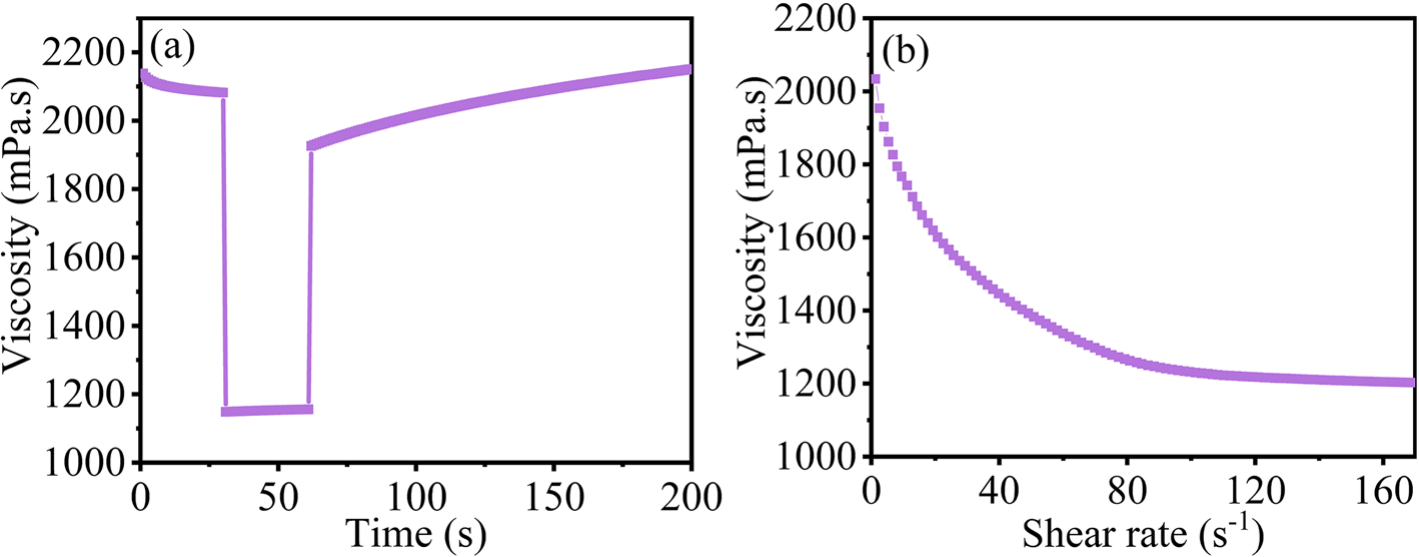
(**a**) The rheological properties of the ZnO QDs water-based fluorescent ink during screen printing (25 °C). (**b**) The shear properties of the ZnO QDs water-based fluorescent ink.

The rheology is a technical parameter to measure ink thixotropy and suitability of printing methods. Based on the principle of reducing the amount of organic reagent as much as possible, water-based fluorescent ink was prepared with the PVP content of 0.15 g/mL, M-ZnO QDs concentration of 4%, water as the solvent and pH value of 7.5. In Fig. 6a, the changes in viscosity of the ink at three different stages were measured in order to simulate the ink rheological behaviour during screen printing^42^. In the first stage, the shear rate is 0.1 s^−1^ for 30 s. The viscosity of the ink stabilizes at about 2100 mPa s. In the second stage, the shear rate increases to 200 s^−1^ for 30 s to simulate the squeegee process during screen printing. The viscosity of the ink drops to 1150 mPa s, ensuring the ink smoothly passed through the screen mesh. In the third stage, the shear rate is reduced to 0.1 s^−1^ for 200 s to simulate the recovery of viscosity after the ink is transferred to the print substrate. When the ink squeegee process has finished, the viscosity of the ink gradually recovers to the initial value at 90 s. In this process, the ink is evenly distributed and formed the printed film. Finally, the viscosity of the ink recovers to 2150 mPa s at 140 s, which is slightly greater than the initial viscosity, probably due to the evaporation of water. As a typical non-Newtonian fluid, ink usually has the shear-thinning phenomena. As shown in Fig. 6b, as the shear rate increases, the viscosity of the ink gradually decreases. Until the shear rate has been greater than 80 s^−1^, the viscosity stabilizes at 1200 mPa s. The above studies indicate that the QDs ink is suitable for screen printing.

**Figure 7:**
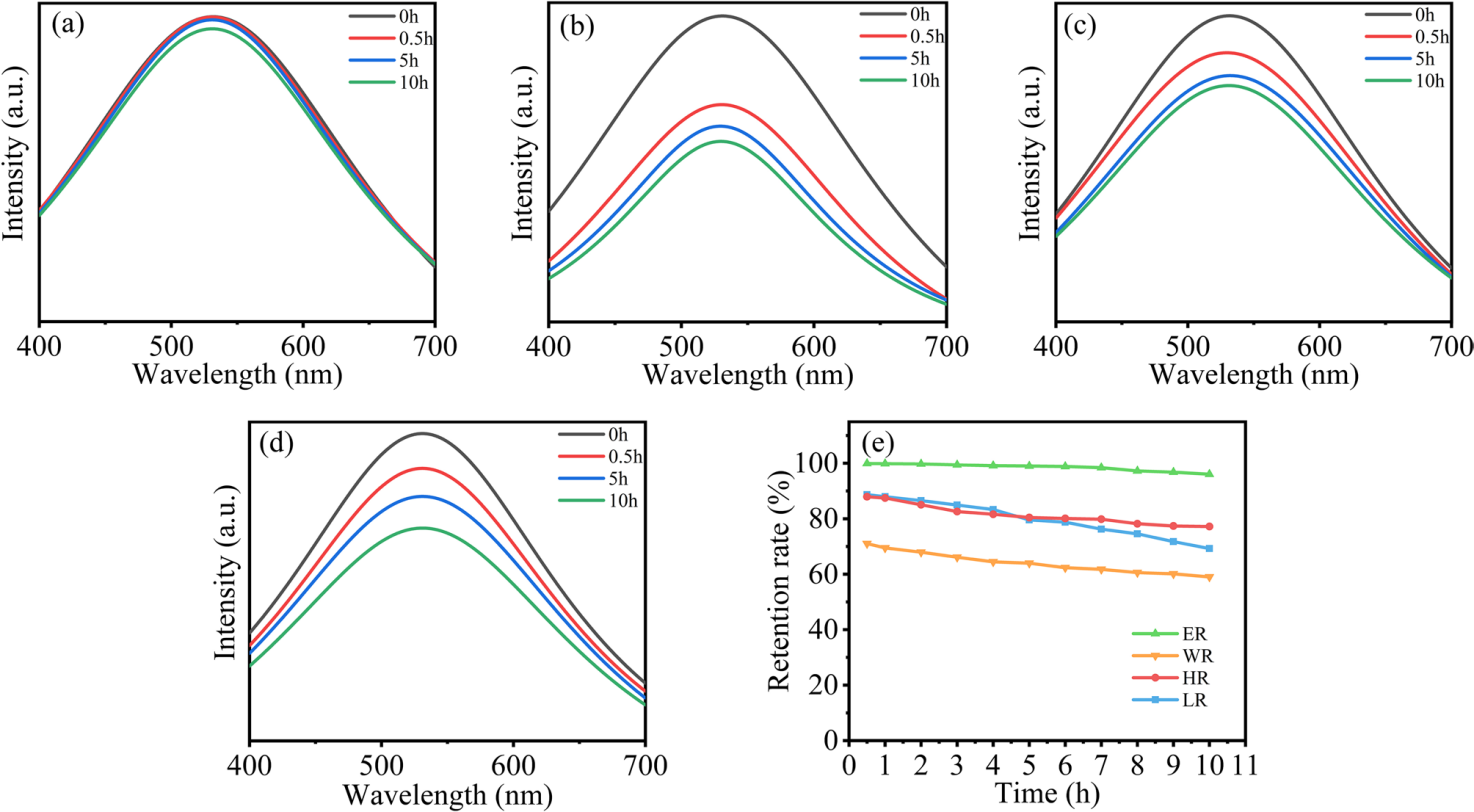
(**a**) The fluorescence spectra of the samples soaked in absolute ethanol for 0 h, 0.5 h, 5 h and 10 h. (**b**) The fluorescence spectra of the samples immersed in hot water at 80 °C for 0 h, 0.5 h, 5 h and 10 h. (**c**) The fluorescence spectra of the samples placed in a 100 °C oven for 0 h, 0. 5 h, 5 h and 10 h. (**d**) The fluorescence spectra of the samples irradiated with UV light for 0 h, 0.5 h, 5 h and 10 h. (**e**) The trends of the fluorescence retention rate of the ZnO QDs water-based fluorescent ink resistance.

The fluorescence is the most important property of fluorescent anti-counterfeiting ink, so it is necessary to test the resistance of the ink fluorescence under extreme conditions. The screen-printed samples of the ZnO QDs water-based fluorescent ink were tested for the four types of resistance. Figure 7a–d show the fluorescence spectra of light resistance (LR), heat resistance (HR), ethanol resistance (ER) and hot water resistance (WR). Combined with Fig. 7e, it can be observed that the ZnO QDs ink has good ER. After being soaked for 10 h, the fluorescence of the sample still retains more than 96%. The WR is slightly poor. After 0.5 h of immersion, the fluorescence intensity has remained 71.03%, and after 10 h, the fluorescence intensity remains 59.02%. The HR and LR are at a medium level. At the initial 0.5 h, the fluorescence retention rates are about 90%. After 10 h, the HR is slightly better than the LR.

### Anti-counterfeiting method of fluorescent ink

**Figure 8:**
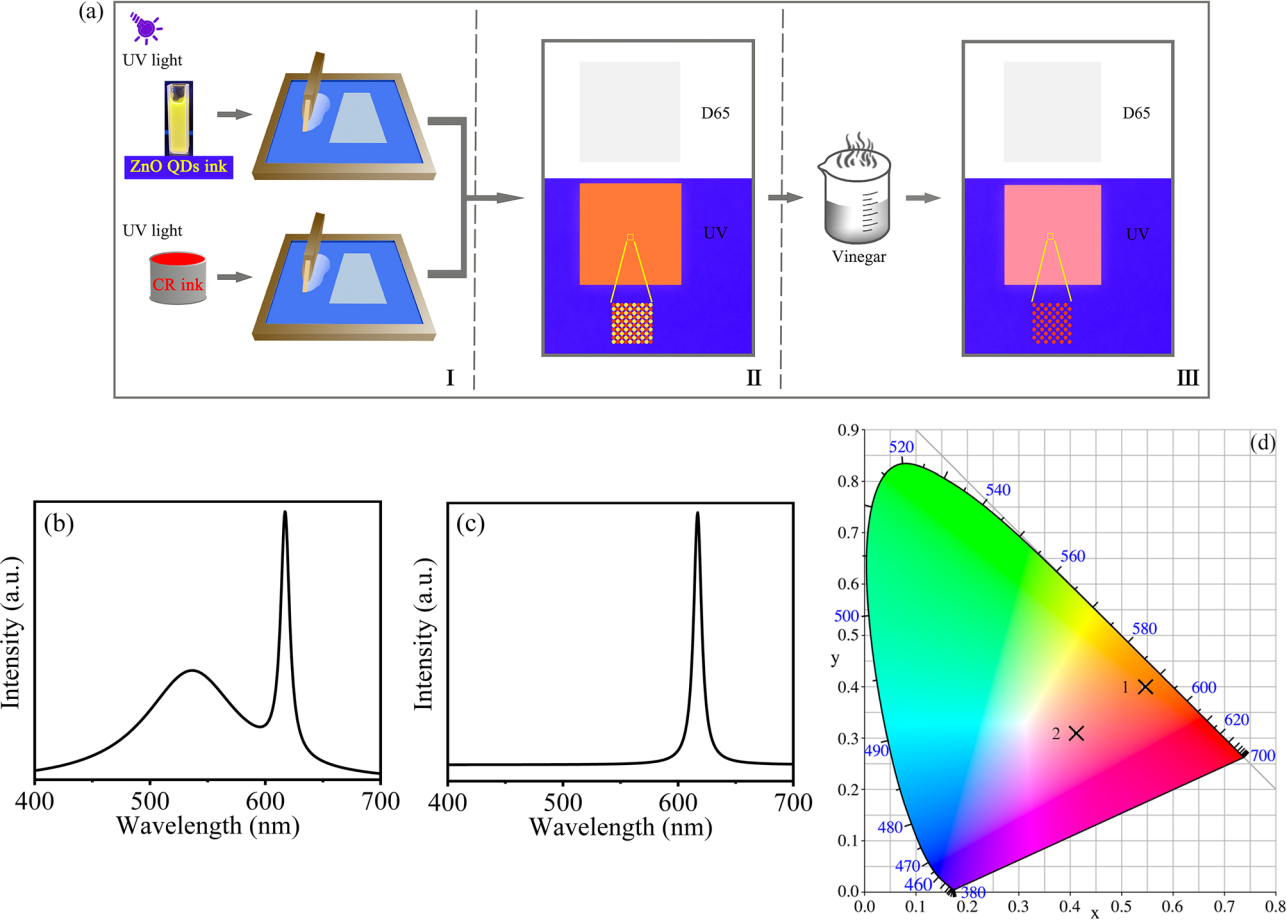
(**a**) The flow chart of the fluorescent discoloration anti-counterfeiting method. (**b**) The fluorescence spectrum of the overprinted color patch before acid treatment. (**c**) The fluorescence spectrum of the overprinted color patch after acid treatment. (**d**) The CIE1931 color coordinates of two color patches before and after acid treatment.

Combining organic fluorescent ink and ZnO QDs fluorescent ink can further improve the anti-counterfeiting function of fluorescent ink. As illustrated in Fig. 8aI, the printing plate is the screen ruling of 200 lpi and the halftone tonal value of 50%. The CR ink and ZnO QDs fluorescent ink were used to overprint. The overprinting effect that the color patch is invisible under D65 light and orange red fluorescence under UV illumination is shown in Fig. 8aII. In addition, the partial enlarged image simulates the arrangement of two kinds of fluorescent ink dots. If the dot percentage of the ink is adjusted, the fluorescence effect of various hues can be obtained and better anti-counterfeiting effect can be achieved. According to the fluorescence quenching of the ZnO QDs by acid described above, the color patch was treated by vinegar in Fig. 8aIII, the color patch does not change under D65 light and only emits red fluorescence from CR ink under UV illumination, which implies that the yellow fluorescence of self-made QDs fluorescent ink is destroyed. The action further enhances the security of the method.

The fluorescence spectrum of the overprinted color patch before acid treatment is shown in Fig. 8b. There are two obvious peaks at 536 nm and 617 nm, which belong to the ZnO QDs ink and the CR ink respectively. In Fig. 8c, only one peak is at 617 nm and the intensity is unchanged after acid treatment. The spectral data have been converted to CIE1931 color coordinate in Fig. 8d. The coordinate 1 (0.5456, 0.3696) before acid treatment and coordinate 2 (0.4105, 0.3097) after acid treatment have apparent difference and can be used for anti-counterfeiting identification.

This method takes advantage of halftone technology to present a variety of mixed fluorescent colors. Then in virtue of the acid resistance of the organic fluorescent ink and the acid quenching of the ZnO QDs ink, the multiple anti-counterfeiting functions of fluorescent ink are realized. The method not only fully reflects the optical anti-counterfeiting properties of the ZnO QDs, but also combines the printing technology to make itself anti-copy, improving the overall security.

## Conclusions

Using APTES as the surface modifier, the ZnO QDs were prepared by the sol–gel method with an average particle size of 7.4 nm, good crystallization, water solubility and light stability, which provided the possibility for preparing the ZnO QDs water-based fluorescent ink. When the pH value is 7.5, the aqueous solution with 4% M-ZnO QDs has the best fluorescence performance. And when the content of PVP is 0.15–0.17 g/mL, the stable ZnO QDs water-based fluorescent ink formulation is formed. The ink is translucent and white under D65 light and emits strong yellow fluorescence under UV illumination. It has rheology suitable for screen printing and great ethanol resistance. In addition, this paper proposes an anti-counterfeiting method of fluorescent discoloration using halftone technology. The effectiveness of this method is achieved from several aspects, such as the anti-replication of micro-dots, the variability of dot percentage, and the significant discoloration before and after acid treatment. The ZnO QDs fluorescent ink is environmentally friendly and can be widely used in the fields of food, drug and cosmetics anti-counterfeiting packaging, personalized printing packaging. In the futher study, we will explore how to improve the fluorescence intensity and stability of ZnO QDs further, and continue to optimize the ink formulation to improve the QDs ink properties and make it suitable for flexographic printing, digital inkjet printing.

## Data Availability

The data generated and analysed during the current study will be made available from the corresponding author on reasonable request.
